# Legionellosis Outbreak Associated with Asphalt Paving Machine, Spain, 2009

**DOI:** 10.3201/eid1609.100248

**Published:** 2010-09

**Authors:** Mireia Coscollá, José Fenollar, Isabel Escribano, Fernando González-Candelas

**Affiliations:** Author affiliations: Universidad de Valencia/Instituto Cavanilles de Biodiversidad y Biología Evolutiva, Valencia, Spain (M. Coscollá, F. González-Candelas);; Centro de Investigación Biomédica en Red en Epidemiología y Salud Pública, Barcelona, Spain (M. Coscollá, F. González-Candelas);; Swiss Tropical and Public Health Institute, Basel, Switzerland (M. Coscollá);; Centro Salud Pública, Alcoi, Spain (J. Fenollar);; Hospital Virgen de los Llirios, Alcoi (I. Escribano)

**Keywords:** Legionella pneumophila, sequence-based typing, outbreak, Legionnaires’ disease, legionellosis, milling machine, natural spring water, bacteria, Spain, research

## Abstract

From 1999 through 2005 in Alcoi, Spain, incidence of legionellosis was continually high. Over the next 4 years, incidence was lower, but an increase in July 2009 led health authorities to declare an epidemic outbreak. A molecular epidemiology investigation showed that the allelic profiles for all *Legionella pneumophila* samples from the 2009 outbreak patients were the same, thus pointing to a common genetic origin for their infections, and that they were identical to that of the organism that had caused the previous outbreaks. Spatial-temporal and sequence-based typing analyses indicated a milling machine used in street asphalt repaving and its water tank as the most likely sources. As opposed to other machines used for street cleaning, the responsible milling machine used water from a natural spring. When the operation of this machine was prohibited and cleaning measures were adopted, infections ceased.

## Abstract 2

TOC Summary: The source was untreated spring water in the machine’s water tank.

*Legionella pneumophila* (*1*) is a gram-negative bacterium identified as the causative agent of an outbreak of pneumonia that occurred in a Philadelphia hotel during a Legionnaires’ convention in 1977 (*2*). This outbreak affected 221 persons, of whom 34 died (*2*). Although other *Legionella* spp. can cause the disease, *L. pneumophila* is responsible for 90% of the cases of legionellosis globally. This species is 1 of the most common causes of community-acquired bacterial pneumonia (*3**–**7*) and the second most common cause of severe pneumonia (*8*).

*L. pneumophila* is a waterborne bacterium that can cause respiratory illness when a susceptible person inhales contaminated, aerosolized water. Infection sources are usually human-made aquatic habitats, such as potable water supplies (*9*), whirlpool spas (*10*), cooling towers (*11**,**12*), showers (*13*), decorative fountains (*14**,**15*), and hoses (*16*).

Molecular epidemiologic analyses of *L. pneumophila* usually compare sequence-based typing patterns from bacterial cultures derived from putative environmental sources with *L. pneumophila* cultures from patients’ sputum samples. This approach consists of amplifying and sequencing internal fragments of 7 loci and assigns a number to the different alleles derived from each locus (*17**,**18*). The combination of allele variants for each locus determines the allelic profile that characterizes each sample. Isolation of *L. pneumophila* from respiratory secretions on selective media is fastidious and time-consuming. Moreover, some *L. pneumophila* strains may be viable but cannot be cultured (*19*); despite high specificity of this method, culturing *L. pneumophila* is not efficient (*20**,**21*). Therefore, the efficiency of sequence-based typing of *L. pneumophila* can be improved by direct amplification and sequencing of DNA extracted from uncultured respiratory samples (*22*).

Since 2000, a specific epidemiologic surveillance system for legionellosis has been in place in the Hospital Virgen de los Llirios in Alcoi, Spain. Every patient with signs of pneumonia is scanned by chest radiography and urine analysis for *L. pneumophila* serogroup 1 antigen. This surveillance system enables early detection and better prognosis for *L. pneumophila*–infected patients. It also helps distinguish between sporadic cases and outbreaks, thus enabling early start of epidemiologic investigations.

At the end of July 2009, the epidemiology surveillance system detected 2 cases of legionellosis in persons who had stayed in Alcoi, Spain, during their incubation periods. New cases appeared during the first week of August, at which time an epidemic outbreak was declared and an epidemiologic investigation was started. Patients in the outbreak were questioned about clinical and personal aspects. Spatial–temporal analysis was used to identify the most likely areas of exposure for infection. *L. pneumophila* was isolated from environmental samples obtained in those areas. Because the usual facilities and municipal water systems associated with risk were not contaminated, other facilities not previously linked to legionellosis outbreaks were considered. To verify the common genetic origin of the outbreak and its environmental source, we performed an epidemiologic molecular analysis using sequence-based typing for clinical and environmental samples.

## Materials and Methods

### Legionellosis Cases

Cases of pneumonia reported in Alcoi during the incubation period were considered suspected cases of legionellosis unless this diagnosis could be ruled out. A confirmed case of legionellosis was defined as a case of pneumonia with laboratory evidence of acute infection with *L. pneumophila* including 1) isolation of *L. pneumophila* serogroup 1 from respiratory secretions or lung tissue, 2) a >4-fold rise in antibody titers (from 128) against *L. pneumophila* serogroup1 by immunofluorescence in paired acute- and convalescent-phase serum specimens, or 3) detection of *L. pneumophila* serogroup 1 antigen in urine.

Cases that conformed to the case definition and/or had positive results in at least 1 of the following tests were considered suspected cases: 1) high (>256) antibody titer against *L. pneumophila* serogroup 1 in convalescent-phase serum, 2) seroconversion to *L. pneumophila* serogroup 1 by indirect inmunofluorescence in acute- and convalescent-phase serum, or 3) direct staining of bacteria in respiratory secretions or lung tissue by direct fluorescence using monoclonal or polyclonal antibodies against any *Legionella* spp. or serogroup, including serogroup 1.

### Epidemiologic Investigation

A questionnaire was used to obtain personal information (age, gender, job, and free-time activities), clinical features (signs and symptoms, date of illness onset, date of hospitalization, previous hospitalizations, and medical history), predisposing and risk factors, place of residence, and recent urban mobility within Alcoi. Information about the patients’ addresses and usual itineraries (roads and places visited) in Alcoi was used to delimit an area of influence, or a buffer, for the location of the likely source of infection. A 500-m radius was considered around home and city itineraries for each patient by using vectorial cartography (scale 1:10,000), ortophotography (scale 1:5,000), and ArcView software (www.esri.com). The buffer for each patient was thus represented by a polygonal area; intersecting areas yielded a common area representing a likely location for the source of the outbreak.

### Environmental Investigation

Systematic environmental investigations are regularly performed in the municipal water distribution system, but when this outbreak was detected, an active search for *L. pneumophila* was made in patients’ homes (bulk water and biofilms from showerheads and taps) and the water distribution system (bulk water); results were negative. None of the other usual sources (e.g., public fountains, cooling towers, humidifiers) were found to pose a risk. The absence of usual risk sources led us to consider other possible sources of aerosols, including moving devices used in street cleaning and asphalt repaving; the latter had been observed in the risk area during the epidemiologic inspection.

### Laboratory Methods

#### Clinical Sampling

Respiratory samples and, when available, corresponding cultures were obtained from 11 patients with a diagnosis of legionellosis made by positive urine test result at the Hospital Virgen de los Lirios. DNA was extracted from respiratory isolates by using an UltraClean BloodSpin Kit (Mobio Laboratories, Inc., Carlsbad, CA, USA). DNA from positive *L. pneumophila* cultures was extracted as described (*23*). Briefly, bacterial colonies from pure cultures were resuspended in 200 μL of 20% Chelex 100 resin (Bio-Rad Laboratories, Richmond, CA, USA). DNA was then extracted during 3 freeze–thaw cycles (–75°C for 10 min and 94°C for 10 min), and cellular debris was removed by centrifuging at 10,000 × *g* for 1 min. The amount of genomic DNA was measured by spectrophotometry at 260 nm in triplicate, and DNA purity was checked by using the A260/A280 ratio. Purified DNA was stored at –20°C until used. DNA extraction from respiratory samples (sputum, bronchoalveolar aspirated secretions, and lung biopsy tissue) was performed with a QIAamp DNA Mini Kit (QIAGEN, Valencia, CA, USA) and stored at –20°C.

#### Environmental Sampling

Water samples were filtered and treated with acid. Water and swab specimens were plated onto buffered charcoal yeast extract medium with and without supplemental antimicrobial drugs; standard plating techniques were used. Inoculated plates were incubated at 35°C and examined regularly for colonies resembling *Legionella* spp. Suspected colonies were inoculated onto biplates containing buffered charcoal yeast extract medium with and without l-cysteine. Nine cultures that required l-cysteine for growth were subcultured and tested with specific antiserum to determine the *Legionella* spp. and serogroup.

### PCR, Sequencing, and Allelic Profile Assignment

For cultured isolates, the 7 loci in the European Working Group for *Legionella* Infections (EWGLI, www.ewgli.org) typing scheme were amplified as detailed by Gaia et al. (*17*) and Ratzow et al. (*18*). DNA from respiratory samples was amplified by using a seminested approach. For the first PCR we used the same primers and conditions as for cultured isolates. For the second PCR we used the internal primers described by Coscollá et al. (*22*) and used 2 μL of the first PCR products as template. Amplification conditions and profiles were the same as for the first PCR except for the annealing temperature (*22*). PCR products were purified by using a High Pure PCR Product Purification Kit (Roche Diagnostics, Mannheim, Germany). PCRs were conducted in a reaction volume of 50 μL containing 20 ng of genomic DNA, 1 U of Taq DNA polymerase, 200 μmol/L of each dNTP, 2.5 mmol/L of magnesium-free buffer, 2.5 mmol/L MgCl_2_, and 0.2 μmol/L of each pair of primers. We adopted the gene notation used in the first *L. pneumophila* genome published (*24*); consequently, locus *fliC* corresponds to locus *flaA* used in the EWGLI typing scheme (*17*).

Purified DNA was directly sequenced by the dideoxy method by using a BigDye Terminator v3.0 Ready Reaction Cycle Sequencing Kit and was analyzed in an ABI PRISM 3700 sequencer (each from Applied Biosystems, Foster City, CA, USA). Sequencing of PCR products from respiratory samples differed only in the use of the same internal primers used in the second PCR of the seminested approach. Sequence chromatogram files were analyzed by using the Staden sequence analysis package (*25*).

Allelic profiles for the 7 sequenced genes were obtained from EWGLI and were aligned and compared with sequences derived in this study. Multiple sequence alignments were obtained by using ClustalX (*26*) and further refined by visual inspection.

### Molecular Phylogenetic Analysis

A phylogenetic reconstruction was obtained with the concatenated alignment of sequences from the 7 loci analyzed. Models of nucleotide substitution were assessed by using the maximum-likelihood approach implemented in jModeltest (*27*). Maximum-likelihood phylogenetic trees were obtained with PHYML 3.0 (*28*) by using the previously derived models of nucleotide substitution for each locus. Support for the nodes was evaluated by bootstrapping with 1,000 pseudoreplicates.

## Results

### Epidemiologic Findings

Among patients with positive urine antigen test results, 11 cases of legionellosis were confirmed and *L. pneumophila* was isolated from 4. All patients required hospitalization, and all except 1 recovered. (The patient who did not recover had severe signs and symptoms and subsequently died.) The main signs and symptoms were fever (100% incidence), pneumonia (100%), headache (27.3%), myalgia (27.3%), diarrhea and/or vomiting (18.2%), and confusion (45.5%). The average age was 70 years, range 49–88 years. More men than women were affected (male:female ratio = 4.5).

**Figure 1:**
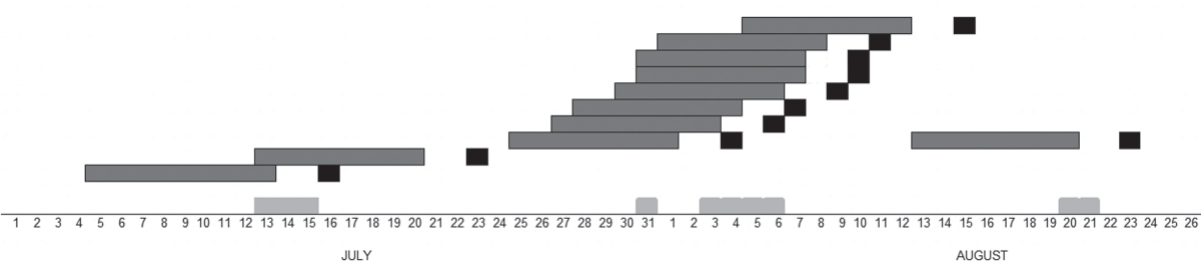
Timeline for epidemic of legionellosis, Alcoi, Spain, 2009. Light gray squares indicate days the paving machine was working; dark gray squares indicate incubation period; black squares indicate day of illness onset.

Confirmed cases occurred from July 21 through September 17. According to the date of disease onset, the outbreak showed 3 epidemic waves: 2 cases in the second half of July, 8 cases in the first half of August, and 1 case in the second half of August (Figure 1).

**Figure 2:**
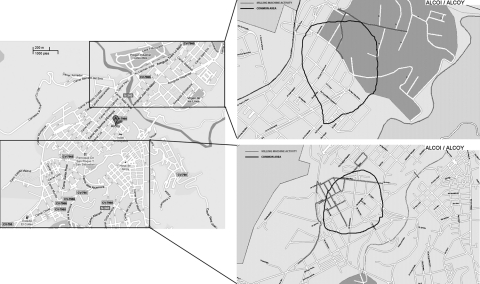
The 2 neighborhoods in Alcoi, Spain, with identified risk areas for the outbreak of legionellosis, 2009 (left). For each area, an enlarged map at right shows the common risk area (black lines) and the streets where the milling machine and water tank had operated during the incubation periods (gray lines).

No common indoor source of exposure was found, and the initial hypothesis was that the outbreak originated from environmental contamination of an unknown source capable of producing and dispersing large quantities of aerosols contaminated with *L. pneumophila*. The first 2 patients lived in the northern part of the city, which suggested that the source could be located in that area. The spatial distribution of patients’ buffers changed in August, thus indicating that the likely source of the outbreak had moved to the Santa Rosa quarter (Figure 2). The area of epidemic risk was modified accordingly, and the search for putative environmental sources focused on that neighborhood.

### Environmental Findings

*L. pneumophila* was not isolated from samples derived from the municipal water supply, traditional risk facilities, and patients’ houses. The environmental investigation was extended to other potential sources, especially portable cleaning devices such as sweepers, hydrocleaners, and water tanks used to clean the streets, all of which used water from the municipal water supply. At that time, the Santa Rosa quarter was being repaved, and 1 of the machines used in the repaving process was a tank truck that carried water used by a large milling machine. The water in the tank was obtained from a natural spring untreated with chlorine or anything else. Because the average daytime temperature in Alcoi during July and August is 27°C, the water in the machine might have been warm enough for *L. pneumophila* growth. The milling machine had been working north of Alcoi around July 15 and in the Santa Rosa neighborhood from July 31 through August 20 (Figure 2). This activity fits spatially and temporally with the incubation period of confirmed cases. This machine was identified and removed from service on August 21, thus was able to cause the last infection detected on August 23 (Figure 2).

### Microbiological Findings

**Figure 3:**
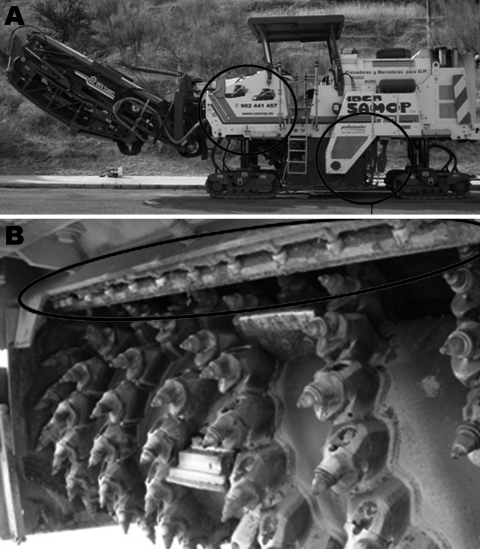
Milling machine and water tank used for asphalt paving in Alcoi, Spain, 2009, during outbreak of legionellosis. The 2,000-L water tank (A, left circle) supplied the 18 atomizers (A, right circle; B, oval), which sprayed 8,000 L water/d.

*L. pneumophila* serogroup 1 was isolated from the water in the tank and from the atomizers in the milling machine (Figure 3). *L. pneumophila* was also isolated from other machines used for street cleaning in the city, but these organisms were from serogroups other than serogroup1.

The milling machine and the water tank were immediately confined outside the city, sealed, cleaned, and disinfected. When the machine was put back into use, a new cleaning and maintenance protocol was implemented to prevent future contamination. The cleaning protocol consisted of treatment with chlorine (20 ppm) and removal of the atomizers and their replacement by gravity-based water distributors. Additionally, for operation in the city, use of a separate thermo-insulated water tank in which chlorine was continuously applied at 20 ppm was required, thus preventing any further growth of *L. pneumophila*. Additionally, water had to be obtained from the municipal system.

### Molecular Characteristics

*L. pneumophila* sequence information was obtained from 7 uncultured sputum samples and from 4 cultures derived from the 11 patients studied. Complete allelic profiles, according to the EWGLI typing scheme, were obtained for 9 clinical samples in the study. All had the same profile, which corresponded to sequence type (ST) 578 in the EWGLI database (Table 1). Partial allelic profiles for the other 2 samples were consistent with ST578. The 9 *L. pneumophila* isolates derived from putative environmental sources, including the milling machine and the water tank (Table 2), were also characterized and corresponded to 4 STs: ST578 in 4 samples, ST1 in 3 samples, ST777 in 1 sample, and a new ST in sample 4159.

**Table 1:** Sequence information for *Legionella pneumophila* isolates from patients with legionellosis, Alcoi, Spain, August 2009*

Patient no.	Sex	Sample collection date	Sample type	EWGLI sequence-based typing pattern	ST
0203	M	13	Isolate and sputum	6, 10, 15, 13, 9, 14, 6	578
0205	F	13	Sputum	6, 10, 15, 13, 9, 14, 6	578
0260	F	17	Sputum	6, 10, 15, 13, 9, 14, 6	578
0263	F	17	Isolate and sputum	6, 10, 15, 13, 9, 14, 6	578
0293	M	18	Sputum	6, 10, 15, 13, 9, 14, 6	578
0295	M	18	Isolate and sputum	6, 10, 15, 13, 9, 14, 6	578
0300	M	18	Sputum	0, 0, 15, 0, 9, 0, 0	NA
0351	F	20	Sputum	6, 0, 15, 0, 9, 0, 0	NA
0353	M	20	Sputum	6, 10, 15, 13, 9, 14, 6	578
0372	M	20	Sputum	6, 10, 15, 13, 9, 14, 6	578
1107	F	30	Isolate and sputum	6, 10, 15, 13, 9, 14, 6	578

*EWGLI, European Working Group for *Legionella* Infections; ST, sequence type; 0, sequence not available; NA, sequence type not available.

**Table 2:** *Legionnella pneumophila* isolated during environmental investigation, Alcoi, Spain, 2009*

Isolate no.	Sampling site	EWGLI sequence-based typing pattern	ST
4143	Cold-water tap (sports club)	1,4,3,1,1,1,1	1
4145	Water accumulator (sports club)	6,10,15, 13, 9,14,6	578
4159	Street sweeper (sprayer)	1,10,14,10,18, X†, 0‡	New
4160	Street sweeper (drain tap)	1,4,3,1,1,1,1	1
4161	Street sweeper (container)	5,2,22,10,6,25,1	777
7968	Water tank (hose pipe)	6,10,15, 13, 9,14,6	578
7969	Milling machine (tap 1)	6,10,15, 13, 9,14,6	578
7970	Milling machine (tap 2)	1,4,3,1,1,1,1	1
7973	Water tank	6,10,15, 13, 9,14,6	578

*EWGLI, European Working Group for *Legionella* Infections; ST, sequence type. †Not obtained. ‡Unassigned new allele.

**Figure 4:**
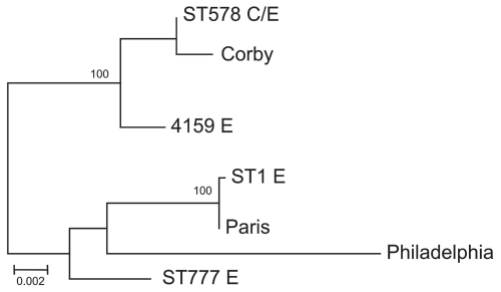
Maximum-likelihood phylogenetic tree obtained from the concatenated alignment of 7 *Legionella pneumophila* genome loci obtained from clinical (C) and environmental (E) samples during outbreak of legionellosis in Alcoi, Spain, 2009. Isolates with identical sequence types (Table 1, Table 2) are represented as 1 isolate. Parentheses enclose the number of samples showing each sequence type. Reference sequences for Philadelphia, Paris, and Corby strains are included. Nodes supported by bootstrap values >70% are indicated. Scale bar indicates 0.002 nucleotide substitutions/position in the sequence.

A phylogenetic tree was obtained from the concatenated alignment of the 2,984 bp (Figure 4). The phylogenetic analysis showed that 4 environmental samples were identical to the clinical samples (represented by ST578 C/E in Figure 4). Their comparison with the remaining environmental samples showed 14–50 nt differences to strains 4159 and 4143/4160/7970 (represented by ST1 in Figure 4), respectively. The comparison of these to reference strains showed that outbreak samples were more closely related to the Corby strain (*29*), a virulent human isolate from which it differed by only 6 nt.

## Discussion

During the epidemic outbreak of legionellosis in the summer of 2009 in Alcoi, Spain, 11 affected persons were identified, and their *L. pneumophila* isolates shared the same sequence-based typing profile (ST578). Spatial-temporal analysis of outbreak cases pointed to a milling machine used in street asphalt repaving and its water tank as the most likely source of infection. Molecular typing confirmed that *L. pneumophila* isolated from the machine showed the same allelic profile as the samples from the patients. When this machine was removed from service and cleaned, infections in this locality ceased.

The absence of apparent risk facilities in the area during the outbreak period and the changing spatial distribution of cases led us to consider alternative sources of contamination and spread. The heterogeneous spatial grouping led us to hypothesize that the transmission sources could be mobile. The analysis of the water tank and milling machine used in both neighborhoods where risk areas were identified during the incubation period of confirmed cases resulted in isolation of *L. pneumophila*. Molecular results confirmed that 4 of the 9 environmental isolates obtained showed a sequence-based typing profile identical to that of all clinical samples. This result highlights the fact that a device not previously considered to represent a risk for *L. pneumophila* infection, such as a street paving machine, can be associated with a legionellosis outbreak.

These kinds of machines are good candidates for spreading *L. pneumophila* infections, given their ability to generate aerosols. These machines are used in urban areas where contaminated aerosols can be inhaled by many citizens. They are continually moving, making their identification as a source of infection more difficult because by the time an outbreak is detected and the causative *L. pneumophila* strains are characterized, these machines have usually moved to another location. Lack of suitable cleaning routines for these machines makes them excellent candidates for colonization with *L. pneumophila*. In this particular outbreak, use of untreated water from a natural spring contributed to the contamination of the tank and milling machine. These devices are frequently stored and operated in and from industrial areas where nontreated water supplies are common, which increases risk for colonization by *L. pneumophila*. Whether they spread *L. pneumophila* more easily than other devices usually linked to such outbreaks, like cooling towers or spas, is unknown, but they should be considered risk devices for the reasons detailed above.

During 1999–2005, ST578 has been found in clinical samples from patients with legionellosis in Alcoi (*22*) and has caused recurrent outbreaks and sporadic cases of community-acquired pneumonia. However, to our knowledge, no identical isolate has been found in the environment in this area (M. Coscollá et al., unpub. data). Finding ST578 in a sports club (Table 2), not related to the outbreak, indicates that it can occasionally be found in other risk facilities, which would help to explain its association with past clinical cases in Alcoi. Additionally, this profile was reportedly found in an environmental strain detected in Mexico during an epidemiologic investigation of travel-associated legionellosis (EWGLI sequence-based typing database, www.ewgli.org).

Although culture isolates were available for only 4 of the 11 outbreak patients identified, our use of a sequence-based typing approach to analyze clinical samples on the basis of direct extraction and sequencing of *L. pneumophila* from sputum samples increased the number of patients we were able to study (i.e., all those identified in the outbreak). The efficiency of this approach has been demonstrated to be higher than that of sequencing after isolating *L. pneumophila* from cultures (*22*). Moreover, the 100% match between the sequences directly obtained from respiratory samples and cultured isolates from the same patient corroborates the suitability of the direct sequencing approach for the identification and molecular epidemiology studies of *L. pneumophila* (*22*). We think that this approach extends the usefulness of molecular epidemiologic tools in the study of *L. pneumophila* outbreaks, thus enabling more precise identification of their sources.
